# The association between sex hormones and periodontitis among American adults: A cross-sectional study

**DOI:** 10.3389/fendo.2023.1125819

**Published:** 2023-02-14

**Authors:** Xingyang Su, Kun Jin, Xianghong Zhou, Zilong Zhang, Chichen Zhang, Yifan Li, Mi Yang, Xinyi Huang, Shishi Xu, Qiang Wei, Xu Cheng, Lu Yang, Shi Qiu

**Affiliations:** ^1^ Department of Urology, Institute of Urology and National Clinical Research Center for Geriatrics, West China Hospital of Sichuan University, Chengdu, China; ^2^ Department of Urology, the First Affiliated Hospital of Zhejiang University, Hangzhou, China; ^3^ West China School of Public Health and West China Fourth Hospital, Sichuan University, Chengdu, China; ^4^ Division of Endocrinology and Metabolism, West China Hospital, Sichuan University, Chengdu, China; ^5^ State Key Laboratory of Oral Diseases, West China School of Stomatology, Sichuan University, Chengdu, China; ^6^ Center of Biomedical big data, West China Hospital, Sichuan University, Chengdu, China

**Keywords:** sex hormones, bioavailable testosterone, periodontitis, NHANES, SHBG

## Abstract

**Introduction:**

After adulthood, as a person grows older, the secretion of sex hormones in the body gradually decreases, and the risk of periodontitis increases. But the relationship between sex hormones and periodontitis is still controversial.

**Methods:**

We investigated the association between sex hormones and periodontitis among Americans over 30 years old. 4,877 participants containing 3,222 males and 1,655 postmenopausal females who had had periodontal examination and detailed available sex hormone levels, were included in our analysis from the 2009-2014 National Health and Nutrition Examination Surveys cycles. We applied multivariate linear regression models to estimate the connection between sex hormones and periodontitis after converting sex hormones into categorical variables through tertile. Additionally, to ensure the stability of the analysis results, we carried out a trend test, subgroup analysis, and interaction test.

**Results:**

After fully adjusting the covariates, estradiol levels were not associated with periodontitis in both males and females with a P for trend = 0.064 and 0.064, respectively. For males, we found that sex hormone-binding globulin was positively associated with periodontitis (tertile3 vs tertile1: OR=1.63, 95% CI=1.17-2.28, p = 0.004, P for trend = 0.005). Congruously, free testosterone (tertile3 vs tertile1: OR=0.60, 95% CI=0.43-0.84, p = 0.003), bioavailable testosterone (tertile3 vs tertile1: OR=0.51, 95% CI=0.36-0.71, p < 0.001), and free androgen index (tertile3 vs tertile1: OR=0.53, 95% CI=0.37-0.75, p < 0.001) was found to be negatively associated with periodontitis. Moreover, subgroup analysis of age found a closer relationship between sex hormones and periodontitis in those younger than 50 years.

**Conclusion:**

Our research suggested that males with lower bioavailable testosterone levels affected by sex hormone-binding globulin were at a higher risk of periodontitis. Meanwhile, estradiol levels were not associated with periodontitis in postmenopausal women.

## Introduction

By 2100, the global population of those older than 65 years is anticipated to reach 2.37 billion according to the Global Burden of Disease Study (1). The estimated global average prevalence of severe periodontitis is about 11%, where the prevalence of severe periodontitis in adults and the elderly is at a minimum 16.9% and possibly up to 48.0% (2). Disability-adjusted life-years (DALYs) for oral health problems due to population aging increased by 28.02% (95% UI 25.87-30.09) in the 20 years from 1990 to 2010 (3). The 2019 Global Burden of Disease study indicated that oral diseases, including severe periodontitis (chronic gum disease), cause 23.1 million DALYs all over the world (4). Management of periodontitis is highly relevant in light of a growing aging population cohort and with the consequential disease burden increasing.

Periodontitis is a chronic, microbial-associated, host-mediated inflammatory disease of the oral cavity (5). The inflammation initiated and driven by a dysbiosis of bacterial biofilm contributes to the progressive destruction of the dental supporting tissues (6), including the loss of periodontal ligaments and alveolar bone (2). Several modifiable risk factors have been established, such as poor oral hygiene, cigarette smoking, and poorly controlled diabetes mellitus (7). Studies have also found that there are many potential risk factors for periodontitis, such as metabolic syndrome, alcohol consumption, osteoporosis, a low intake of calcium and vitamin D, obesity (8–12), and hyperuricemia (13). Interestingly, sex hormones are associated with many of the diseases or factors mentioned above.

Sex hormones are important endocrine substances in the human body that are related to many functions, such as growth, reproduction, and differentiation. Testosterone (T) is a primary male sex hormone synthesized from cholesterol in Leydig cells in males, and estradiol (E2), another of the sex hormones, is mainly derived from the ovaries in females. But E2 is also derived from the conversion of androgens by aromatase in adipose tissue in males and adrenal glands and ovaries also produce T in females. Low T and E2 are connected with a series of medical disorders including obesity, metabolic syndrome, diabetes, cardiovascular disease, and osteoporosis (14–18). Sex hormones play a powerful role in the regulation of inflammatory response and bone turnover through cytokine regulation and direct actions on osteoblast and osteoclast precursors (19–21). A study of male rats found that induced periodontitis in low and high testosterone groups resulted in increased alveolar bone loss compared to a control group (22). Meanwhile, both low T and E2 levels and an elevated incidence of periodontitis are associated with aging (23–25). Based on the association, it is plausible that low sex hormone levels could be associated with periodontitis.

The prevalence estimate of periodontitis depends on the case definition of periodontitis and methods of periodontal surveillance (26). There are some differences between the full mouth periodontal examination (FMPE) protocol and the partial mouth periodontal examination (PMPE) protocol for periodontal examination. Though both protocols exclude the third molars, the FMPE protocol comprehensively measures all the teeth and measures more positions per tooth compared to the PMPE protocol. It is more appropriate to detect periodontitis cases using the FMPE protocol instead of the PMPE protocol because partial-mouth protocols underestimate the prevalence of periodontitis (2, 27, 28). As our investigation sought to assess associations between serum sex steroid levels and periodontitis in an age cohort beyond 30 years, our database necessarily included datasets from the National Health and Nutrition Examination Survey (NHANES) from 2009-2014 (28).

## Methods

### Data source and participants

The data that we used for the analysis were derived from the NHANES. The Centers for Disease Control and Prevention (CDC) conducts the NHANES in the United States *via* questionnaires and examinations in a two-year cycle, with the aim of establishing a database of the health and nutritional status of the US population. As a cross-sectional survey with a complex, multistate, probabilistic sampling design, the NHANES ensures that nationally representative data are available. The National Center for Health Statistics (NCHS) institutional Review Board examines and gives permission to the study and each participant in the survey signs the appropriate written permission. More elaborate information on the methods and protocol of the NHANES is available on the CDC’s official website.

**Figure 1:**
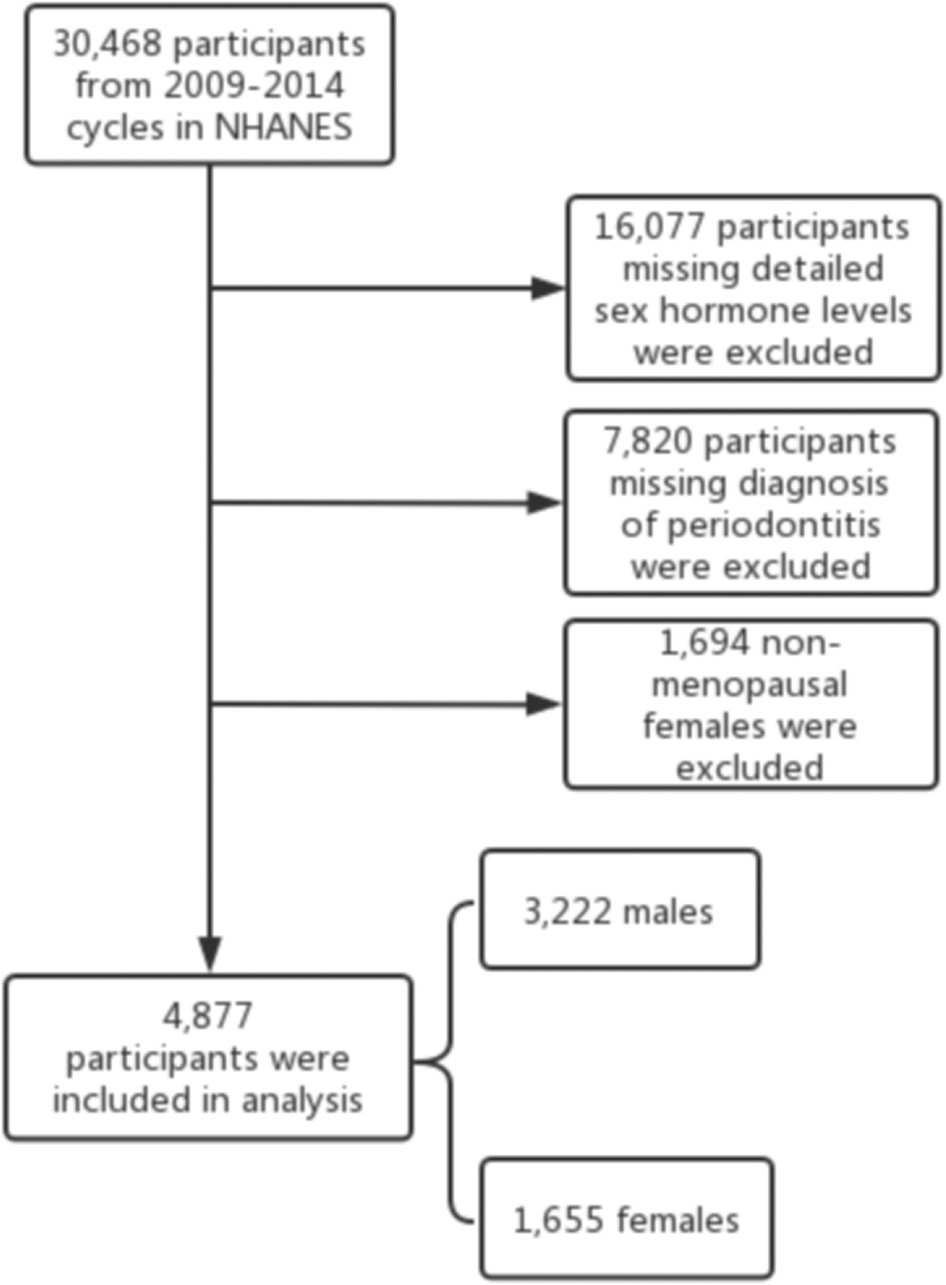
Flow chart of inclusion and exclusion criteria for our analysis.

30,468 participants from the 2009-2010, 2011-2012, and 2013-2014 cycles were selected. The study population was restricted to participants with detailed sex hormone levels and a diagnosis of periodontitis. Considering the dramatic changes in hormone levels during the female physiological cycle, we chose only postmenopausal women who answered “no” to the question “Have you had at least one menstrual period in the past 12 months?” and answered “hysterectomy” or “menopause/life changes” to the question “What is the reason why you have not had a period in the last 12 months?” in the self-reported reproductive health questionnaire (29). Finally, 4,877 participants were included in the analysis, containing 3,222 males and 1,655 females. The inclusion and exclusion flow of the analyzed objects was demonstrated in **Figure 1**.

### Sex hormones

Serum total T (TT) and E2 levels were measured using an isotope dilution high-performance liquid chromatography-tandem mass spectrometry (ID-LC-MS/MS) in the NHANES, while the concentrations of sex hormone-binding globulin (SHBG) were quantified using chemo-luminescence measurements of the reaction products *via* a photomultiplier tube after binding to SHBG with immuno-antibodies (30). According to previous literature, we only indirectly assessed the approximate amount of circulating free testosterone (FT) (31) and the activity of aromatase (32) through the free androgen index (FAI) that was calculated as the value of TT (ng/dL) divided by SHBG (nmol/L) and the ratio of TT to E2 (TT/E2), respectively. Bioavailable testosterone was calculated according to the Vermeulen et al. formula (33).

### Periodontitis

The FMPE that included clinical attachment loss (AL) and probing depth (PD) was used for the periodontal examination from 2009 to 2014. The dental examiner used the HU-Friedy periodontal probe to perform the assessment. AL and PD were assessed at six positions per tooth using a perioprobe for a maximum of up to 28 teeth (2, 34). Cases of periodontitis were determined according to the suggested CDC-AAP definitions which were used for monitoring periodontitis. Periodontitis requires the patient’s periodontal measurement of ≥ 2 inter-proximal sites with ≥ 3 mm AL and ≥ 2 inter-proximal sites with ≥ 4 mm PD on different teeth or one site with ≥ 5 mm (27). In this study, the severity of periodontitis was not differentiated. The absence of any symptoms of periodontitis described above was defined as the absence of periodontitis.

### Covariates

Covariates associated with periodontitis and T were collected, including some demographic information such as age, race, ratio of family income to poverty, educational level, and civil state. All demographic data could be obtained from questionnaire data and they were converted into the corresponding categorical variables. Smoking status was classified as never, former, and current, respectively, and alcohol intake per day was classified as never, moderate, and heavy, respectively. Time of venipuncture, BMI, diabetes, and hypertension were also potential confounding factors. White blood cells (WBC), which partly reflect inflammation in an individual’s body, were also adjusted in the analysis (35–37).

### Statistical analysis

We used the statistical software package R (http://www.Rproject.org,theRFoundation) and Empower (R) to carry out all the analysis, and a p-value of ≤ 0.05 was required to be statistically significant. In the data analysis, continuous variables and categorical variables were denoted by mean ± SD/Median (Q1-Q3) and proportions, respectively. A sample weight was assigned to each participant because of the multistate, probabilistic sampling design (38). For categorical variables and continuous variables, we performed a weighted chi-square test and a Kruskal Wallis test to calculate characteristics of otherness between male and female groups, respectively. Our purpose was to probe the relationship between sex hormones and periodontitis, so we used weighted multivariate linear regression models in different sex groups after sex hormones tertiles. Multivariate models included the non-adjusted model, adjusted model (only age, race, BMI, ratio of family income to poverty, education level, time of venipuncture, civil state, smoking status, alcohol intake per day, and WBC level were adjusted), and adjusted model II (diabetes and hypertension were adjusted additionally). Simultaneously, we carried out a trend test to confirm the stability of the results. Finally, we used weighted stratified line regression models for subgroup analysis of age. Some categorical covariables were used to perform an interaction test. We used interaction terms between subgroup indicators to test the effect modification in subgroups, followed by a likelihood-ratio test.

## Results

Sex hormone levels and covariates of the male and female periodontitis participants in this study are characterized in **Table 1**. The prevalence of periodontitis in males was 59.5%, obviously higher than that in females (34.2%). The population characteristics were not consistent in age, race, BMI, ratio of family income to poverty, education level, time of venipuncture, civil state, smoking status, alcohol intake per day, and also WBC level, diabetes, and hypertension in the two groups of participants: that is, there were statistical differences. Compared with the female groups, the male groups were more likely to be older, married, or living with a partner, a former or current smoker, and have diabetes and hypertension, as well as having a lower WBC level, a higher poverty-to-income ratio, a lower education level, a higher BMI, and a higher alcohol intake per day. It was not surprising that participants in the male groups had higher TT levels, lower E2 levels, lower SHBG levels, higher FT levels, higher FAI levels, and higher bioavailable testosterone levels. **Table S1** shows the comparison of sex hormone levels between groups with and without periodontitis. There was no significant difference in testosterone and estradiol levels between the periodontitis group and the non-periodontitis group in males or females. In males, the periodontitis group had higher SHBG levels and lower bioavailable testosterone levels than the non-periodontitis group. Inversely, the periodontitis group had a lower SHBG level than the non-periodontitis group in females.

**Table 1:** Baseline characteristics of NHANES participants between 2009 and 2014 in male and female groups (n = 4877).

Gender	Male	Female	Standardize diff.	P-value
Number	3222	1655		
Age, years, mean (SD)	51.99 (14.38)	42.31 (10.32)	0.77 (0.71, 0.83)	<0.001
Testosterone, nmol/L, median (Q1-Q3)	1.07 (0.81-1.42)	0.06 (0.04-0.08)	2.92 (2.84, 3.00)	<0.001
Estradiol, pg/mL, median (Q1-Q3)	23.40 (18.20-29.10)	66.50 (26.20-129.00)	0.96 (0.87, 1.04)	<0.001
SHBG, nmol/L, median (Q1-Q3)	38.59 (27.89-54.05)	65.74 (44.88-98.10)	0.79 (0.70, 0.88)	<0.001
free testosterone, nmol/L, median (Q1-Q3)	0.02 (0.01-0.02)	0.00 (0.00-0.00)	3.74 (3.60, 3.88)	<0.001
Bioavailable testosterone, nmol/L, median (Q1-Q3)	0.41 (0.33-0.50)	0.02 (0.01-0.02)	3.55 (3.41, 3.69)	<0.001
Free androgen index	2.81 (2.17-3.62)	0.09 (0.06-0.14)	2.57 (2.46, 2.69)	<0.001
WBC, 10^9/L, mean (SD)	7.01 (2.11)	7.33 (2.24)	0.15 (0.09, 0.21)	<0.001
Race, n (%)			0.10 (0.04, 0.16)	0.02
Mexican American	411 (12.76%)	220 (13.29%)		
Other Hispanic	285 (8.85%)	153 (9.24%)		
Non-Hispanic White	1333 (41.37%)	623 (37.64%)		
Non-Hispanic Black	691 (21.45%)	346 (20.91%)		
Other Race	502 (15.58%)	313 (18.91%)		
Ratio of family income to poverty, n (%)			0.11 (0.05, 0.17)	<0.001
<1.3	862 (26.75%)	527 (31.84%)		
1.3-3.5	1008 (31.28%)	485 (29.31%)		
>3.5	1352 (41.96%)	643 (38.85%)		
Education level, n (%)			0.10 (0.04, 0.16)	0.005
Less than high school	669 (21.27%)	315 (19.61%)		
High school or GED General educational development	678 (21.56%)	296 (18.43%)		
Above high school	1798 (57.17%)	995 (61.96%)		
Civil state, n (%)			0.10 (0.04, 0.16)	0.001
Married or living with partner	2301 (71.44%)	1108 (66.99%)		
Living alone	920 (28.56%)	546 (33.01%)		
BMI, n (%)			0.28 (0.22, 0.34)	<0.001
<=25	828 (25.82%)	543 (33.09%)		
25-30	1307 (40.75%)	454 (27.67%)		
>30	1072 (33.43%)	644 (39.24%)		
Smoking status, n (%)			0.47 (0.41, 0.53)	<0.001
Never	1515 (47.05%)	1135 (68.58%)		
Former	1134 (35.22%)	290 (17.52%)		
Current	571 (17.73%)	230 (13.90%)		
Alcohol intake per day, n (%)			0.32 (0.26, 0.38)	<0.001
None	2293 (71.17%)	1348 (81.45%)		
Moderate	390 (12.10%)	67 (4.05%)		
Heavy	539 (16.73%)	240 (14.50%)		
Diabetes, n (%)			0.23 (0.17, 0.29)	<0.001
No	2693 (86.12%)	1505 (93.02%)		
Yes	434 (13.88%)	113 (6.98%)		
Hypertension, n (%)			0.29 (0.23, 0.35)	<0.001
No	1998 (62.09%)	1246 (75.38%)		
Yes	1220 (37.91%)	407 (24.62%)		
Time of venipuncture, n (%)			0.07 (0.01, 0.13)	0.049
Morning	1582 (49.10%)	798 (48.22%)		
Afternoon	1172 (36.37%)	573 (34.62%)		
Evening	468 (14.53%)	284 (17.16%)		
Periodontitis, n (%)			0.52 (0.46, 0.58)	<0.001
No	1304 (40.47%)	1089 (65.80%)		
Yes	1918 (59.53%)	566 (34.20%)		

**Table 2** shows the association of sex hormone levels with periodontitis in males after sorting by tertile. The results of our analysis showed no significant relationship between TT levels and periodontitis (tertile3 vs tertile1: OR=0.91, 95% CI=0.73–1.13, p = 0.384, P for trend = 0.491). E2 levels were not associated with periodontitis (tertile2 vs tertile1: OR=0.80, 95% CI=0.61-1.07, p = 0.131; tertile3 vs tertile1: OR=0.75, 95% CI=0.56–1.01, p = 0.060). The trend test gave a similar result (P for trend = 0.064). For SHBG, we found that SHBG (tertile3 vs tertile1: OR=1.63, 95% CI=1.17-2.28, p = 0.004) was positively associated with periodontitis with P for trend = 0.005. Congruously, FT (tertile3 vs tertile1: OR=0.60, 95% CI=0.43-0.84, p = 0.003), bioavailable testosterone (tertile3 vs tertile1: OR=0.51, 95% CI=0.36-0.71, p < 0.001), and FAI (tertile3 vs tertile1: OR=0.53, 95% CI=0.37-0.75, p < 0.001) was found to be negatively associated with periodontitis with P for trend = 0.004, < 0.001, and < 0.001, respectively.

**Table 2:** The association of sex hormone levels sorted by tertile with periodontitis in males. .

Exposure	Non-adjusted	Adjusted I	Adjusted II
Testosterone, ng/dL, OR (95%CI) P-value			
T1 (1.44 - 314.08)	1	1	1
T2 (314.39 - 448.38)	0.86 (0.72, 1.02) 0.084	0.83 (0.68, 1.01) 0.069	0.82 (0.67, 1.01) 0.061
T3 (448.41 - 2543.99)	1.04 (0.87, 1.24) 0.658	0.90 (0.73, 1.12) 0.345	0.91 (0.73, 1.13) 0.384
P for trend	1.00 (1.00, 1.00) 0.503	1.00 (1.00, 1.00) 0.428	1.00 (1.00, 1.00) 0.491
Estrodiol, pg/mL, OR (95%CI) P-value			
TI (2.12 - 20.00)	1	1	1
T2 (20.10 - 27.00)	0.77 (0.60, 0.97) 0.030	0.79 (0.60, 1.04) 0.091	0.80 (0.61, 1.07) 0.131
T3 (27.10 - 95.10)	0.91 (0.71, 1.15) 0.430	0.77 (0.58, 1.03) 0.078	0.75 (0.56, 1.01) 0.060
P for trend	0.99 (0.98, 1.01) 0.496	0.98 (0.97, 1.00) 0.086	0.98 (0.96, 1.00) 0.064
SHBG, nmol/L, OR (95%CI) P-value			
TI (6.90 - 31.41)	1	1	1
T2 (31.42 - 48.06)	1.59 (1.24, 2.05) 0.0003	1.28 (0.95, 1.73) 0.101	1.26 (0.93, 1.71) 0.139
T3(48.08 - 196.50)	2.47 (1.90, 3.21) <0.0001	1.66 (1.20, 2.31) 0.002	1.63 (1.17, 2.28) 0.004
P for trend	1.02 (1.02, 1.03) <0.0001	1.01 (1.00, 1.02) 0.003	1.01 (1.00, 1.02) 0.005
Free testosterone, ng/dL, OR (95%CI) P-value			
TI (5.35e-05 - 0.015362954)	1	1	1
T2 (0.015384232 - 0.019664324)	0.62 (0.48, 0.81) 0.0004	0.71 (0.52, 0.95) 0.023	0.74 (0.54, 1.00) 0.052
T3 (0.01966649 - 0.103391716)	0.46 (0.35, 0.59) <0.0001	0.57 (0.41, 0.80) 0.001	0.60 (0.43, 0.84) 0.003
P for trend	0.00 (0.00, 0.00) <0.0001	0.00 (0.00, 0.00) 0.001	0.00 (0.00, 0.00) 0.004
Bioavailable testosterone, ng/dL, OR (95%CI) P-value			
TI (0.001091759 - 0.355356499)	1	1	1
T2 (0.355702816 - 0.464128567)	0.64 (0.49, 0.83) 0.0007	0.74 (0.55, 1.00) 0.052	0.75 (0.55, 1.02) 0.068
T3 (0.464900842 - 2.638546259)	0.38 (0.29, 0.50) <0.0001	0.49 (0.35, 0.68) <0.0001	0.51 (0.36, 0.71) 0.0001
P for trend	0.02 (0.01, 0.07) <0.0001	0.06 (0.02, 0.22) <0.0001	0.07 (0.02, 0.27) <0.0001
Free androgen index, OR (95%CI) P-value			
TI (0.006991234 - 2.4220154)	1	1	1
T2 (2.4225 - 3.27821137)	0.60 (0.46, 0.78) 0.0001	0.67 (0.49, 0.90) 0.009	0.68 (0.50, 0.93) 0.017
T3 (3.2828038 - 32.33420366)	0.33 (0.25, 0.42) <0.0001	0.49 (0.35, 0.69) <0.0001	0.53 (0.37, 0.75) 0.0004
P for trend	0.59 (0.53, 0.67) <0.0001	0.72 (0.62, 0.85) <0.0001	0.75 (0.64, 0.88) 0.001

Non-adjusted model adjusted for: none; Adjusted I model adjusted for: age, race, BMI, time of venipuncture, ratio of family income to poverty, education level, civil state, smoking status, alcohol intake per day, and WBC level; Adjusted II model adjusted for: age, race, BMI, time of venipuncture, ratio of family income to poverty, education level, civil state, smoking status, alcohol intake per day, WBC level, diabetes, and hypertension; T, tertile.

**Table 3** shows the association of sex hormone levels with periodontitis in females after sorting by tertile. Similar to males, we found no significant relationship (tertile3 vs tertile1: OR=1.00, 95% CI=0.82-1.22, p = 0.984, P for trend = 0.963) between TT level and periodontitis. E2 levels were also not associated with periodontitis in females (tertile2 vs tertile1: OR=0.67, 95% CI=0.50-0.90, p = 0.009; tertile3 vs tertile1: OR=0.59, 95% CI=0.41-0.86, p = 0.005; P for trend = 0.064). However, no significant relationship was observed between SHBG and periodontitis in women (tertile3 vs tertile1: OR=1.16, 95% CI=0.85-1.58, p = 0.354, P for trend = 0.508).

**Table 3:** The association of sex hormone levels sorted by tertile with periodontitis in females.

Exposure	Non-adjusted	Adjusted I	Adjusted II
Testosterone, ng/dL, OR (95%CI) P-value			
T1 (1.02 - 14.28)	1	1	1
T2 (14.3 - 22.88)	0.86 (0.72, 1.01) 0.069	1.05 (0.86, 1.27) 0.649	1.04 (0.86, 1.26) 0.690
T3 (22.9 - 575.0)	0.78 (0.66, 0.92) 0.004	1.00 (0.82, 1.22) 0.997	1.00 (0.82, 1.22) 0.984
P for trend	0.99 (0.98, 1.00) 0.005	1.00 (0.99, 1.01) 0.934	1.00 (0.99, 1.01) 0.963
Estradiol, pg/mL, OR (95%CI) P-value			
T1 (2.117 - 7.25)	1	1	1
T2 (7.26 - 49.20)	0.59 (0.47, 0.75) <0.0001	0.67 (0.50, 0.90) 0.007	0.67 (0.50, 0.90) 0.009
T3 (49.3 - 1220)	0.38 (0.29, 0.48) <0.0001	0.59 (0.41, 0.85) 0.005	0.59 (0.41, 0.86) 0.005
P for trend	0.99 (0.99, 0.99) <0.0001	1.00 (0.99, 1.00) 0.061	1.00 (0.99, 1.00) 0.064
SHBG, nmol/L, OR (95%CI) P-value			
T1 (9.20 - 47.82)	1	1	1
T2 (47.86 - 79.86)	1.15 (0.90, 1.46) 0.274	1.28 (0.96, 1.71) 0.090	1.27 (0.95, 1.70) 0.103
T3 (79.90 - 758.8)	0.89 (0.70, 1.14) 0.365	1.17 (0.86, 1.59) 0.322	1.16 (0.85, 1.58) 0.354
P for trend	1.00 (0.99, 1.00) 0.238	1.00 (1.00, 1.01) 0.472	1.00 (1.00, 1.01) 0.508

Non-adjusted model adjusted for: none; Adjusted I model adjusted for: age, race, BMI, time of venipuncture, ratio of family income to poverty, education level, civil state, smoking status, alcohol intake per day, and WBC level; Adjusted II model adjusted for: age, race, BMI, time of venipuncture, Ratio of family income to poverty, education level, civil state, smoking status, alcohol intake per day, WBC level, diabetes, and hypertension; T, tertile.

It is well known that the occurrence of periodontitis is closely associated with age, and the subgroup analysis of age is shown in **Tables 4**, **5**. In adjusted model II, there was a weak association between testosterone and periodontitis in participants aged younger than 50 years in females (P for trend = 0.046). Interestingly, we found that subjects with higher SHBG levels were more likely to develop periodontitis in males younger than 50 years old (tertile2 vs tertile1: OR=1.48, 95% CI=0.99-2.20, p = 0.056; tertile3 vs tertile1: OR=1.84, 95% CI=1.11-3.07, p = 0.019; P for trend = 0.014), while this phenomenon was not present in males older than 50 years. At the same time, as shown in **Tables S2**, **S3**, we did not detect a significant interaction regarding the correlation between periodontitis and sex hormones.

**Table 4:** The subgroup analysis of age for the association of sex hormone levels and periodontitis in males.

Exposure	Age <50 years	Age >=50 years	Total
Adjusted II			
Testosterone, ng/dL, OR (95%CI) P-value			
T1 (1.44 - 314.08)	1	1	1
T2 (314.39 - 448.38)	0.82 (0.62, 1.09) 0.180	0.81 (0.61, 1.09) 0.161	0.82 (0.67, 1.01) 0.061
T3 (448.41 - 2543.99)	0.96 (0.70, 1.32) 0.824	0.81 (0.59, 1.10) 0.172	0.91 (0.73, 1.13) 0.384
P for trend	1.00 (1.00, 1.00) 0.955	1.00 (1.00, 1.00) 0.200	1.00 (1.00, 1.00) 0.491
Estradiol, pg/mL, OR (95%CI) P-value			
T1 (2.117 - 20)	1	1	1
T2 (20.1 - 27)	0.73 (0.49, 1.08) 0.119	0.87 (0.58, 1.33) 0.531	0.80 (0.61, 1.07) 0.131
T3 (27.1 - 95.1)	0.69 (0.44, 1.07) 0.101	0.76 (0.50, 1.15) 0.192	0.75 (0.56, 1.01) 0.060
P for trend	0.98 (0.95, 1.00) 0.100	0.98 (0.96, 1.01) 0.191	0.98 (0.96, 1.00) 0.064
SHBG, nmol/L, OR (95%CI) P-value			
T1 (6.9 - 31.41)	1	1	1
T2 (31.42 - 48.06)	1.48 (0.99, 2.20) 0.056	0.96 (0.58, 1.57) 0.863	1.26 (0.93, 1.71) 0.139
T3 (48.08 - 196.5)	1.84 (1.11, 3.07) 0.019	1.31 (0.80, 2.15) 0.279	1.63 (1.17, 2.28) 0.004
P for trend	1.02 (1.00, 1.03) 0.014	1.01 (1.00, 1.02) 0.144	1.01 (1.00, 1.02) 0.005

Adjusted II model adjusted for: age, race, BMI, time of venipuncture, Ratio of family income to poverty, education level, civil state, smoking status, alcohol intake per day, WBC level, diabetes, and hypertension; T, tertile.

**Table 5:** The subgroup analysis of age for the association of sex hormone levels and periodontitis in females.

Exposure	Age <50 years	Age >=50 years	Total
Adjusted II			
Testosterone, ng/dL, OR (95%CI) P-value			
T1 (1.79 - 16.84)	1	1	1
T2 (16.85 - 25.7)	1.15 (0.83, 1.58) 0.403	1.97 (0.98, 3.95) 0.056	1.23 (0.92, 1.64) 0.155
T3 (25.71 - 575)	0.76 (0.54, 1.06) 0.102	1.24 (0.60, 2.57) 0.569	0.84 (0.62, 1.12) 0.234
P for trend	0.98 (0.97, 1.00) 0.046	1.01 (0.98, 1.04) 0.555	0.99 (0.98, 1.00) 0.121
Estradiol, pg/mL, OR (95%CI) P-value			
T1 (2.117 - 37.5)	1	1	1
T2 (37.6 - 101)	0.79 (0.50, 1.25) 0.319	1.11 (0.18, 6.83) 0.914	0.79 (0.52, 1.21) 0.275
T3 (102 - 1220)	0.93 (0.58, 1.47) 0.745	1.55 (0.36, 6.75) 0.560	0.92 (0.60, 1.40) 0.690
P for trend	1.00 (1.00, 1.00) 0.976	1.00 (0.99, 1.01) 0.560	1.00 (1.00, 1.00) 0.893
SHBG, nmol/L, OR (95%CI) P-value			
T1 (9.87 - 50.97)	1	1	1
T2 (51.01 - 85.2)	1.29 (0.81, 2.07) 0.279	1.22 (0.33, 4.43) 0.765	1.29 (0.83, 1.98) 0.255
T3 (85.57 - 758.8)	0.80 (0.48, 1.34) 0.397	1.13 (0.26, 4.95) 0.870	0.93 (0.58, 1.48) 0.754
P for trend	1.00 (0.99, 1.00) 0.278	1.00 (0.98, 1.02) 0.911	1.00 (0.99, 1.00) 0.580

Adjusted II model adjusted for: age, race, BMI, time of venipuncture, Ratio of family income to poverty, education level, civil state, smoking status, alcohol intake per day, WBC level, diabetes, and hypertension; T, tertile.

## Discussion

The target of our study was to explore the connection between sex hormones and periodontitis. This investigation found that TT levels and E2 levels were unrelated to periodontitis, while SHBG levels were significantly positively associated with periodontitis, and FT, FAI, and bioavailable testosterone were all significantly negatively correlated with periodontitis in men.

The relationship between TT and periodontitis is still unclear. Our study found no significant relationship between TT levels and periodontitis and a positive association between SHBG with periodontitis for males. Similar to our study, Orwoll et al. reported the occurrence and progression of periodontitis and tooth loss were independent of serum sex hormone levels among older males over 65 years (35). Consistently, a population-based longitudinal cohort study from Pomerania found no obvious connections between sex hormones with the progression of periodontal measurement or tooth loss (36). However, some studies reported the opposite. Singh et al. reported that the T levels in participants without tooth loss were significantly higher than those in participants with tooth loss in 2011, suggesting T could predict tooth loss very well (39). Androgen deprivation therapy (ADT) is a critical therapeutic option for treating prostate cancer, which is designed to prevent the development of prostate cancer by lowering androgen levels in a patient. Famili et al. spotted that the prevalence of parodontopathy was obviously higher among men on ADT compared with those not on ADT (80.5% vs 3.7%) (40). This paradoxical result makes one wonder if SHBG is where our attention should be. Since T bound to SHBG would lose bioactivity, bioavailable testosterone, including FT and albumin-bound T, could be affected by the level of SHBG. Our conjecture is also supported by the negative correlation between FT, FAI, and bioavailable testosterone and periodontitis in our study. Our results are in concordance with another similar study in 2015 based on the NHANES III, which found that low SHBG was inversely associated with periodontitis (37). This study found that male subjects under the age of 50 with higher SHBG levels were more likely to develop periodontitis (P for trend = 0.014), while those over the age of 50 did not (P for trend = 0.144). Consistently, a cohort study of older adults over 65 years of age found that sex steroid and SHBG levels were not associated with the baseline mean clinical attachment loss and mean PD (35). We currently lack evidence to explain this discrepancy.

T is known to affect bone metabolism through the modulation of immunological events in existing periodontitis (41, 42). A study has found that dihydrotestosterone (DHT) can downregulate the production of IL-6 and upregulate fibroblast proliferation simultaneously (43). Due to lower DHT, the production of the increased proinflammatory cytokine IL-6 by osteoclasts may play a crucial role *via* osteoclastic activity in bone resorption (44). As the predominant cells in periodontal connective tissues, fibroblasts play an important role under inflammatory conditions. In addition, a study showed that T replacement therapy in hypogonadal men significantly induced reductions in serum pro-inflammatory cytokines including TNF and IL-1β (45). T may prevent and control infection of the host by bacteria by increasing expression of E-selectin (induced by TNF-α) and vascular adhesion molecule-1 in endotheliocyte, which are paramount in trans-endothelial migration of phagocytic cells (46, 47). In male rats, testosterone therapy was found to increase the proportion of blood vessels, the extracellular matrix, and fibroblasts in the presence of periodontal inflammation, which may be related to the regulatory effects of PGE2 and IL-10 (48). The decrease in bioavailable testosterone affected by SHBG may be one of the risk factors for the occurrence of periodontitis.

Our study was conducted utilizing the NHANES data, which is representative of the US population and has been obtained using standard protocols and measurements. This ensured that our results had good extrapolation and data reliability. However, this study had some inevitable shortcomings and limitations. Firstly, the cross-sectional nature of the NHANES limited the conclusions about gonadal hormone levels and periodontitis to possible associations, rather than causes. Secondly, serum TT levels were only measured at one time-point in the NHANES data, while American Urological Association (AUA) guidelines recommend two measurements due to intra-individual and diurnal variations of serum T (33). Thirdly, because of the lack of relevant data, such as inflammatory biomarkers, hormone-related drugs, TSH, and free T4, these confounding factors were not adjusted.

## Conclusions

Our research suggested that males with lower bioavailable testosterone levels affected by SHBG were at a higher risk of periodontitis. But we need to carry out a large, carefully designed prospective study for clarifying the causal association between sex hormones and periodontitis due to mechanism complexity.

## Data availability statement

The original contributions presented in the study are included in the article/**Supplementary Material**. Further inquiries can be directed to the corresponding authors.

## Author contributions

Author contributions are as follows: KJ, SQ, QW, and LY contributed equally by conceiving and designing the study. XS, KJ, and SQ organized and analyzed the data. XZ and XS wrote the paper. LY, XC, XZ, ZZ, CZ, YL, MY, XH, and SX revised the manuscript critically for important intellectual content. MY and XH helped to complete the design and evaluation of the statistical methodology in this study. All authors contributed to the article and approved the submitted version.
