# Supplementary material for: The association between sex hormones and periodontitis among American adults: A cross-sectional study
Source: Front Endocrinol (Lausanne). 2023 Feb 14;14:1125819. doi: 10.3389/fendo.2023.1125819 (PMC9971556; doi:10.3389/fendo.2023.1125819)
Supplement: Supplementary file 1 [file Table_1.docx]

Supplementary Material

The association between sex hormones and periodontitis among American adults: a cross-sectional study

Xingyang Su^1^**†**, Kun Jin^1^**†**, Xianghong Zhou^1^**†**, Lu Yang^1*^, Shi Qiu^1,2*^*****

**Correspondence:** Prof. Lu Yang: wycleflue@163.com

Prof. Shi Qiu: [qiushi@scu.edu.cn](mailto:email@uni.eduqiushi@scu.edu.cn)

# 1 Supplementary Figures and Tables

**Table S1: The comparison of sex hormone levels between groups with and without periodontitis**

| **Periodontitis** | **Non-periodontitis group** | **Periodontitis group** | **Standardize diff.** | **P-value** |
| --- | --- | --- | --- | --- |
| **Total** |  |  |  |  |
| Number | 2393 | 2484 |  |  |
| Testosterone, ng/dL, median (Q1-Q3) | 211.24 (22.10-396.00) | 319.40 (143.00-459.00) | 0.39 (0.33, 0.45) | <0.001 |
| Estradiol, pg/mL, median (Q1-Q3) | 28.00 (20.10-58.45) | 24.70 (17.85-34.40) | 0.27 (0.19, 0.35) | <0.001 |
| SHBG, nmol/L, median (Q1-Q3) | 45.13 (29.98-71.02) | 46.04 (31.99-64.84) | 0.14 (0.05, 0.22) | 0.001 |
| Free testosterone, nmol/L, median (Q1-Q3) | 0.01 (0.00-0.02) | 0.01 (0.01-0.02) | 0.25 (0.16, 0.33) | <0.001 |
| Bioavailable testosterone, nmol/L, median (Q1-Q3) | 0.29 (0.02-0.46) | 0.34 (0.14-0.44) | 0.21 (0.12, 0.29) | <0.001 |
| Free androgen index | 0.03 (0.00-0.05) | 0.04 (0.02-0.06) | 0.29 (0.21, 0.37) | <0.001 |
| **Male** |  |  |  |  |
| Number | 1304 | 1918 |  |  |
| Testosterone, ng/dL, median (Q1-Q3) | 377.52 (285.00-482.00) | 370.44 (279.00-503.00) | 0.03 (-0.04, 0.10) | 0.372 |
| Estradiol, pg/mL, median (Q1-Q3) | 23.60 (18.52-28.87) | 23.10 (17.85-29.30) | 0.02 (-0.08, 0.11) | 0.739 |
| SHBG, nmol/L, median (Q1- Q3) | 34.28 (25.00-48.02) | 41.66 (30.42-58.39) | 0.38 (0.27, 0.48) | **<0.001** |
| Free testosterone, nmol/L, median (Q1-Q3) | 0.02 (0.02-0.02) | 0.02 (0.01-0.02) | 0.28 (0.17, 0.38) | **<0.001** |
| Bioavailable testosterone, nmol/L, median (Q1-Q3) | 0.44 (0.36-0.53) | 0.39 (0.31-0.47) | 0.34 (0.23, 0.44) | **<0.001** |
| Free androgen index | 0.05 (0.04-0.06) | 0.05 (0.04-0.06) | 0.06 (-0.03, 0.16) | 0.206 |
| **Female** |  |  |  |  |
| Number | 1089 | 566 |  |  |
| Testosterone, ng/dL, median (Q1-Q3) | 20.92 (14.69-29.77) | 20.29 (14.58-27.58) | 0.09 (-0.01, 0.19) | 0.094 |
| Estradiol, pg/mL, median (Q1-Q3) | 70.50 (31.10-136.00) | 58.80 (18.25-118.00) | 0.14 (-0.00, 0.28) | 0.056 |
| SHBG, nmol/L, median (Q1- Q3) | 69.20 (45.29-104.80) | 59.99 (44.73-89.88) | 0.21 (0.06, 0.36) | **0.009** |
| Free testosterone, nmol/L, median (Q1-Q3) | 0.00 (0.00-0.00) | 0.00 (0.00-0.00) | 0.00 (-0.15, 0.15) | 0.984 |
| Bioavailable testosterone, nmol/L, median (Q1-Q3) | 0.01 (0.01-0.02) | 0.02 (0.01-0.02) | 0.01 (-0.14, 0.16) | 0.877 |
| Free androgen index | 0.00 (0.00-0.00) | 0.00 (0.00-0.00) | 0.15 (0.01, 0.29) | **0.044** |

**Table S2:** **The interaction test for association of sex hormone levels and periodontitis in males**

| **Model** | **Age <50 years** | **Age >=50 years** | **P for interaction** |
| --- | --- | --- | --- |
| Testosterone, ng/dL, OR (95%CI) P-value |  |  |  |
| Crude | 1.0002 (0.9996, 1.0008) 0.5279 | 1.0002 (0.9996, 1.0007) 0.5843 | 0.9220 |
| Model I | 0.9995 (0.9988, 1.0002) 0.1800 | 0.9998 (0.9992, 1.0004) 0.5335 | 0.5402 |
| Model I* | 0.9997 (0.9990, 1.0005) 0.4611 | 0.9996 (0.9989, 1.0002) 0.2028 | 0.7744 |
| Model II | 0.9995 (0.9988, 1.0002) 0.1921 | 0.9999 (0.9992, 1.0005) 0.7309 | 0.4418 |
| Model II* | 0.9997 (0.9990, 1.0005) 0.4995 | 0.9996 (0.9990, 1.0003) 0.2381 | 0.7963 |
| Estradiol, pg/mL, OR (95%CI) P-value |  |  |  |
| Crude | 0.9968 (0.9808, 1.0131) 0.7018 | 1.0001 (0.9863, 1.0141) 0.9861 | 0.7625 |
| Model I | 0.9924 (0.9737, 1.0115) 0.4338 | 0.9938 (0.9780, 1.0099) 0.4502 | 0.9086 |
| Model I* | 0.9882 (0.9688, 1.0080) 0.2398 | 0.9958 (0.9795, 1.0124) 0.6163 | 0.5592 |
| Model II | 0.9898 (0.9710, 1.0090) 0.2957 | 0.9917 (0.9756, 1.0081) 0.3213 | 0.8774 |
| Model II* | 0.9836 (0.9638, 1.0038) 0.1101 | 0.9947 (0.9781, 1.0116) 0.5363 | 0.4018 |
| SHBG, nmol/L, OR (95%CI) P-value |  |  |  |
| Crude | 1.0222 (1.0121, 1.0324) <0.0001 | 1.0064 (1.0000, 1.0128) 0.0514 | 0.0089 |
| Model I | 1.0162 (1.0052, 1.0274) 0.0039 | 1.0065 (0.9990, 1.0141) 0.0877 | 0.1419 |
| Model I* | 1.0160 (1.0047, 1.0273) 0.0053 | 1.0069 (0.9990, 1.0149) 0.0850 | 0.1967 |
| Model II | 1.0150 (1.0040, 1.0262) 0.0077 | 1.0060 (0.9983, 1.0137) 0.1256 | 0.1745 |
| Model II* | 1.0143 (1.0029, 1.0259) 0.0135 | 1.0065 (0.9985, 1.0146) 0.1099 | 0.2730 |

Crude model adjust for: None;

Model I adjust for: race, BMI, time of venipuncture, ratio of family income to poverty, education level, civil state, smoking status, alcohol intake per day and WBC level;

Model I* adjust for: race, BMI, time of venipuncture, ratio of family income to poverty, education level, civil state, smoking status, alcohol intake per day and WBC level and the interaction terms for following variables: BMI, time of venipuncture, race, smoking status;

Model II adjust for: race, BMI, time of venipuncture, Ratio of family income to poverty, education level, civil state, smoking status, alcohol intake per day, WBC level, diabetes and hypertension;

Model II* adjust for: race, BMI, time of venipuncture, Ratio of family income to poverty, education level, civil state, smoking status, alcohol intake per day, WBC level, diabetes and hypertension and the interaction terms for following variables: race, BMI, time of venipuncture, education level, smoking status, alcohol intake per day, WBC level, diabetes and hypertension.

**Table S3: The interaction test for association of sex hormone levels and periodontitis in females**

| **Model** | **Age <50 years** | **Age >=50 years** | **P for interaction** |
| --- | --- | --- | --- |
| Testosterone, ng/dL, OR (95%CI) P-value |  |  |  |
| Crude | 0.9967 (0.9907, 1.0027) 0.2784 | 0.9980 (0.9886, 1.0074) 0.6717 | 0.8205 |
| Model I | 0.9944 (0.9878, 1.0011) 0.1025 | 0.9976 (0.9879, 1.0074) 0.6287 | 0.606 |
| Model I* | 0.9941 (0.9872, 1.0011) 0.0970 | 0.9988 (0.9885, 1.0092) 0.8219 | 0.4734 |
| Model II | 0.9940 (0.9872, 1.0009) 0.0881 | 0.9972 (0.9873, 1.0071) 0.5727 | 0.617 |
| Model II* | 0.9936 (0.9865, 1.0008) 0.0810 | 0.9986 (0.9883, 1.0090) 0.7925 | 0.4504 |
| Estradiol, pg/mL, OR (95%CI) P-value |  |  |  |
| Crude | 0.9989 (0.9972, 1.0007) 0.2418 | 1.0008 (0.9974, 1.0042) 0.6501 | 0.3244 |
| Model I | 0.9993 (0.9972, 1.0013) 0.4840 | 1.0016 (0.9981, 1.0051) 0.3787 | 0.2469 |
| Model I* | 0.9993 (0.9973, 1.0013) 0.5097 | 1.0035 (0.9988, 1.0081) 0.1437 | 0.0709 |
| Model II | 0.9996 (0.9976, 1.0017) 0.7396 | 1.0017 (0.9981, 1.0054) 0.3516 | 0.3077 |
| Model II* | 0.9997 (0.9976, 1.0017) 0.7520 | 1.0040 (0.9987, 1.0092) 0.1380 | 0.0764 |
| SHBG, nmol/L, OR (95%CI) P-value |  |  |  |
| Crude | 0.9954 (0.9919, 0.9989) 0.0092 | 0.9992 (0.9907, 1.0078) 0.8572 | 0.4139 |
| Model I | 0.9976 (0.9941, 1.0012) 0.1881 | 1.0025 (0.9931, 1.0119) 0.6075 | 0.3354 |
| Model I* | 0.9977 (0.9942, 1.0012) 0.1964 | 0.9990 (0.9863, 1.0120) 0.8840 | 0.8433 |
| Model II | 0.9976 (0.9941, 1.0011) 0.1764 | 1.0023 (0.9927, 1.0119) 0.6421 | 0.3583 |
| Model II* | 0.9976 (0.9941, 1.0011) 0.1800 | 0.9981 (0.9850, 1.0113) 0.7760 | 0.9447 |

Crude model adjust for: None;

Model I adjust for: race, BMI, time of venipuncture, ratio of family income to poverty, education level, civil state, smoking status, alcohol intake per day and WBC level;

Model I* adjust for: race, BMI, time of venipuncture, ratio of family income to poverty, education level, civil state, smoking status, alcohol intake per day and WBC level and the interaction terms for following variables: race, BMI, time of venipuncture, , ratio of family income to poverty, education level, civil state and alcohol intake per day;

Model II adjust for: race, BMI, time of venipuncture, Ratio of family income to poverty, education level, civil state, smoking status, alcohol intake per day, WBC level, diabetes and hypertension;

Model II* adjust for: race, BMI, time of venipuncture, Ratio of family income to poverty, education level, civil state, smoking status, alcohol intake per day, WBC level, diabetes and hypertension and the interaction terms for following variables: race, BMI, time of venipuncture, ratio of family income to poverty, education level, civil state, smoking status, alcohol intake per day, diabetes.

**Table S4: The association of sex hormone levels which sorted by tertile with periodontitis in males after adjusting Oral hygiene habits and periodontal treatment history**

| **Exposure** | **Non-adjusted** | **Adjust I** | **Adjust II** |
| --- | --- | --- | --- |
| Testosterone, ng/dL, OR (95%CI) P-value |  |  |  |
| T1 ( 1.44 - 314.08) | 1 | 1 | 1 |
| T2 ( 314.39 - 448.38) | 0.86 (0.72, 1.02) 0.0843 | 0.83 (0.68, 1.02) 0.0702 | 0.82 (0.67, 1.01) 0.0564 |
| T3 (448.41 - 2543.99) | 1.04 (0.87, 1.24) 0.6584 | 0.91 (0.73, 1.12) 0.3690 | 0.90 (0.73, 1.13) 0.3766 |
| P for trend | 1.00 (1.00, 1.00) 0.5029 | 1.00 (1.00, 1.00) 0.4558 | 1.00 (1.00, 1.00) 0.4831 |
| Estrodiol, pg/mL, OR (95%CI) P-value |  |  |  |
| TI (2.12 - 20.00) | 1 | 1 | 1 |
| T2 (20.10 - 27.00) | 0.77 (0.60, 0.97) 0.0300 | 0.77 (0.58, 1.03) 0.0755 | 0.79 (0.59, 1.05) 0.1100 |
| T3 (27.10 - 95.10) | 0.91 (0.71, 1.15) 0.4302 | 0.78 (0.58, 1.05) 0.0972 | 0.76 (0.56, 1.03) 0.0756 |
| P for trend | 0.99 (0.98, 1.01) 0.4961 | 0.98 (0.97, 1.00) 0.1087 | 0.98 (0.96, 1.00) 0.0823 |
| SHBG, nmol/L, OR (95%CI) P-value |  |  |  |
| TI (6.90 - 31.41) | 1 | 1 | 1 |
| T2 (31.42 - 48.06) | 1.59 (1.24, 2.05) 0.0003 | 1.28 (0.95, 1.74) 0.1051 | 1.26 (0.93, 1.72) 0.1368 |
| T3(48.08 - 196.50) | 2.47 (1.90, 3.21) <0.0001 | 1.56 (1.12, 2.18) 0.0094 | 1.55 (1.10, 2.18) 0.0123 |
| P for trend | 1.02 (1.02, 1.03) <0.0001 | 1.01 (1.00, 1.02) 0.0113 | 1.01 (1.00, 1.02) 0.0142 |
| free testosterone, ng/dL, OR (95%CI) P-value |  |  |  |
| TI (5.35e-05 - 0.015362954) | 1 | 1 | 1 |
| T2 (0.015384232 - 0.019664324) | 0.62 (0.48, 0.81) 0.0004 | 0.73 (0.54, 0.99) 0.0462 | 0.75 (0.55, 1.03) 0.0749 |
| T3 (0.01966649 - 0.103391716) | 0.46 (0.35, 0.59) <0.0001 | 0.60 (0.43, 0.84) 0.0029 | 0.62 (0.44, 0.88) 0.0065 |
| P for trend | 0.00 (0.00, 0.00) <0.0001 | 0.00 (0.00, 0.00) 0.0032 | 0.00 (0.00, 0.00) 0.0070 |
| Bioavailable testosterone, ng/dL, OR (95%CI) P-value |  |  |  |
| TI (0.001091759 - 0.355356499) | 1 | 1 | 1 |
| T2 (0.355702816 - 0.464128567) | 0.64 (0.49, 0.83) 0.0007 | 0.76 (0.55, 1.03) 0.0791 | 0.76 (0.55, 1.04) 0.0867 |
| T3 (0.464900842 - 2.638546259) | 0.38 (0.29, 0.50) <0.0001 | 0.52 (0.37, 0.73) 0.0002 | 0.53 (0.38, 0.76) 0.0004 |
| P for trend | 0.02 (0.01, 0.07) <0.0001 | 0.08 (0.02, 0.29) 0.0002 | 0.09 (0.02, 0.34) 0.0004 |
| Free androgen index, OR (95%CI) P-value |  |  |  |
| TI (0.006991234 - 2.4220154) | 1 | 1 | 1 |
| T2 (2.4225 - 3.27821137) | 0.60 (0.46, 0.78) 0.0001 | 0.69 (0.50, 0.94) 0.0202 | 0.70 (0.51, 0.96) 0.0285 |
| T3 (3.2828038 - 32.33420366) | 0.33 (0.25, 0.42) <0.0001 | 0.53 (0.37, 0.75) 0.0003 | 0.55 (0.39, 0.79) 0.0012 |
| P for trend | 0.59 (0.53, 0.67) <0.0001 | 0.75 (0.64, 0.88) 0.0004 | 0.77 (0.65, 0.90) 0.0017 |

Non-adjusted model adjust for: None;

Adjust I model adjust for: age, race, BMI, time of venipuncture, ratio of family income to poverty, education level, civil state, smoking status, alcohol intake per day, WBC level, previous treatment for gum disease, days of dental floss/device use, days of mouthwash use for dental problem, and any teeth loss without an injury;

Adjust II model adjust for: age, race, BMI, time of venipuncture, ratio of family income to poverty, education level, civil state, smoking status, alcohol intake per day, WBC level, diabetes, hypertension, previous treatment for gum disease, days of dental floss/device use, days of mouthwash use for dental problem, and any teeth loss without an injury;

T: tertile

**Table S5: The association of sex hormone levels which sorted by tertile with periodontitis in females after adjusting Oral hygiene habits and periodontal treatment history**

| Exposure | Non-adjusted | Adjust I | Adjust II |
| --- | --- | --- | --- |
| Testosterone, ng/dL, OR (95%CI) P-value |  |  |  |
| T1 (1.79 - 16.84) | 1 | 1 | 1 |
| T2 (16.85 - 25.7) | 1.15 (0.90, 1.47) 0.2763 | 1.28 (0.96, 1.71) 0.0951 | 1.27 (0.95, 1.70) 0.1055 |
| T3 (25.71 - 575) | 0.84 (0.65, 1.08) 0.1775 | 0.86 (0.63, 1.16) 0.3180 | 0.85 (0.63, 1.15) 0.2926 |
| P for trend | 0.99 (0.98, 1.00) 0.1083 | 0.99 (0.98, 1.00) 0.1626 | 0.99 (0.98, 1.00) 0.1484 |
| Estradiol, pg/mL, OR (95%CI) P-value |  |  |  |
| T1 (2.117 - 37.5) | 1 | 1 | 1 |
| T2 (37.6 - 101) | 0.64 (0.45, 0.91) 0.0125 | 0.78 (0.51, 1.20) 0.2559 | 0.79 (0.51, 1.22) 0.2860 |
| T3 (102 - 1220) | 0.65 (0.46, 0.91) 0.0136 | 0.87 (0.57, 1.34) 0.5375 | 0.87 (0.57, 1.34) 0.5326 |
| P for trend | 1.00 (1.00, 1.00) 0.0298 | 1.00 (1.00, 1.00) 0.7172 | 1.00 (1.00, 1.00) 0.6949 |
| SHBG, nmol/L, OR (95%CI) P-value |  |  |  |
| T1 (9.87 - 50.97) | 1 | 1 | 1 |
| T2 (51.01 - 85.2) | 1.13 (0.78, 1.62) 0.5172 | 1.27 (0.82, 1.97) 0.2920 | 1.27 (0.82, 1.98) 0.2847 |
| T3 (85.57 - 758.8) | 0.70 (0.48, 1.03) 0.0692 | 0.89 (0.55, 1.43) 0.6281 | 0.89 (0.56, 1.44) 0.6440 |
| P for trend | 1.00 (0.99, 1.00) 0.0408 | 1.00 (0.99, 1.00) 0.4604 | 1.00 (0.99, 1.00) 0.4734 |

Non-adjusted model adjust for: None;

Adjust I model adjust for: age, race, BMI, time of venipuncture, ratio of family income to poverty, education level, civil state, smoking status, alcohol intake per day, WBC level, previous treatment for gum disease, days of dental floss/device use, days of mouthwash use for dental problem, and any teeth loss without an injury;

Adjust II model adjust for: age, race, BMI, time of venipuncture, ratio of family income to poverty, education level, civil state, smoking status, alcohol intake per day, WBC level, diabetes, hypertension, previous treatment for gum disease, days of dental floss/device use, days of mouthwash use for dental problem, and any teeth loss without an injury;

T: tertile
